# The Regeneration of Divided Nerves

**Published:** 1902-03-22

**Authors:** 


### THE REGENERATION OF DIVIDED NERVES.

The last two meetings of the Royal Medical and Chirurgical Society have been occupied by a very interesting and important discussion on the regeneration of peripheral nerves. On the first occasion Mr. C. A. Ballance read a paper, communicated by himself and Dr. J. Purves Stewart, describing various observations which they had made. There were, he said, two schools of opinion as to the manner of the regeneration. According to one, new nerve fibres grew down from the central end of the divided nerve into the distal segment; according to the other, they were formed in the distal segment itself. The authors supported the latter view, holding that by the proliferation of the neurilemma cells new nerve elements were formed. In a united nerve the neurilemma cells formed new nerve elements, beginning about the end of the third week. In the distal segment of a divided nerve, however, where the ends had been left widely separated, new nerve fibres formed slowly, although they did not attain complete development unless subsequent continuity with the centre were established. Evidently this touched upon the validity of the neuron theory, according to which, as Dr. Purves Stewart pointed out, regeneration of a nerve should not be able to occur in the distal segment of a divided nerve, the ends of which remained widely separated, and therefore the neuron theory broke down in regard to the peripheral nerves. He suggested that the reason why regeneration of nerve fibres in the central nervous system did not occur, was that there were no neurilemma fibres present. Dr. Aldren Turner, however, thought that the neuron theory must not be discarded. There were too many data to support it. There was not only the Wallerian degeneration on the peripheral side and in the cut end of the central segment, but there were also changes which took place in the whole nerve trunk and in the spinal cord. The very statement made by the authors of the paper, to the effect that new fibres never attain maturity until suturing is performed, went to support the neuron theory. Dr. Batten pointed out that although regeneration took place in the stump of the nerve distal to the section, it was certain that, even after three months, it did not reach the finer branches in the muscles, and, if the view of the authors as to the regeneration of nerve by the neurilemma was correct, he thought it would be difficult to explain why regeneration should not take place in the finer nerves within the muscle, just as it was stated to do in the trunk of the nerve. In replying, Dr. Stewart said that the nerves had been examined as far as 7 inches below the lesion, and identically the same changes had been found. The essential fact of their paper was that regeneration occurred in a nerve fibre even when it was permanently cut off from its corresponding nerve cell. The neuron theory could not explain this. It did not fit the facts. It did not explain the absence of regeneration in the central nervous elements, and its presence in the peripheral part of the nervous system. To understand these things it was necessary to take a totally different view of the function of the nerve cell, the purpose of which was evidently to direct or divert the nerve impulses, not to originate them. Thus the central nervous system was to be regarded as a central exchange of nervous impulses. The nerve fibrils were the essential part of the nervous system, the ganglion cells being only what one might call nerve shunts. When an outlying nerve is cut off from the central cell or exchange it is no longer functionally active, and therefore degeneration sets in. But the central part which remains connected with its corresponding nerve cell still receives impulses through it from other fibrils, and so does not degenerate. Later on even the detached outlying fibre becomes regenerated by the activity of the neurilemma cells, and thus is ready to resume its function if it should happen to be linked on to the central nervous system. This view of the matter is something very different from the neuron theory.

